# Selective glucose electro-oxidation catalyzed by TEMPO on graphite felt

**DOI:** 10.3389/fchem.2024.1393860

**Published:** 2024-05-01

**Authors:** Erwann Ginoux, Thibault Rafaïdeen, Patrick Cognet, Laure Latapie, Christophe Coutanceau

**Affiliations:** ^1^ Laboratoire de Génie Chimique, CAMPUS INP-ENSIACET, Toulouse, France; ^2^ Université de Poitiers, CNRS, Institut de Chimie des Milieux et Matériaux de Poitiers IC2MP, Poitiers, France

**Keywords:** glucose, gluconic acid, glucaric acid, optimization, TEMPO, electro-oxidation

## Abstract

Long-term electrolyses of glucose in a potassium carbonate (K_2_CO_3_) aqueous electrolyte have been performed on graphite felt electrodes with TEMPO as a homogeneous catalyst. The influences of the operating conditions (initial concentrations of glucose, TEMPO, and K_2_CO_3_ along with applied anode potential) on the conversion, selectivity toward gluconate/glucarate, and faradaic efficiency were assessed first. Then, optimizations of the conversion, selectivity, and faradaic efficiency were performed using design of experiments based on the L_9_ (3^4^) Taguchi table, which resulted in 84% selectivity toward gluconate with 71% faradaic efficiency for up to 79% glucose conversion. Side products such as glucaric acid were also obtained when the applied potential exceeded 1.5 V vs. reversible hydrogen electrode.

## 1 Introduction

Gluconic acid (GA) has been included in the top-30 list of value-added chemicals derived from biomass (Werpy et al., 2004; Taylor et al., 2015); it has applications in various industries, including food, pharmaceuticals, cosmetics, and construction, and the market for GA is expected to experience sustained growth in the coming decades (Cañete-Rodríguez et al., 2016). At present, biotechnological methods are used to convert glucose into GA (Hustede et al., 2007). While these biotechnological processes have several benefits, such as ecofriendliness, they also have certain drawbacks such as slow conversion rates, requirement for multicomponent media, and complicated separation processes (Tathod et al., 2014). Owing to these limitations, there is growing interest in the development of catalytic methods for transforming glucose into high-value products (Climent et al., 2011; Gallezot, 2012; Tathod et al., 2014). Electrochemical methods for producing GA from glucose were developed quite early (Isabell et al., 1932), and these methods have become more attractive for biomass conversion with the recent increase in renewable electricity production (Li and Sun, 2018; Faverge et al., 2023).

Electrochemical processes have several benefits, including their avoidance of strong chemical oxidants/reductants, high tunability and controllability, and ability to operate at mild temperatures and pressures (Cardoso et al., 2017). In recent times, there have been significant advances in electrocatalysis, resulting in improved energy efficiency and selectivity of electro-organic processes. These achievements involve the use of electrocatalytic materials that reduce the anode and cathode overpotentials, which in turn improve the environmental sustainability of these processes. Numerous studies have been conducted on electrochemical processes using biomass-derived platform molecules (Prabhu et al., 2020; Lucas et al., 2021), such as the electro-oxidation (Latsuzbaia et al., 2018; Taitt et al., 2019) or electroreduction (Lund et al., 1985; Sanghez de Luna et al., 2021) of 5-hydroxymethylfurfural as well as electro-oxidation of glycerol (Zalineeva et al., 2014; Dai et al., 2017; Talebian-Kiakalaieh et al., 2018; Houache et al., 2020), levulinic acid (Qiu et al., 2014; dos Santos et al., 2015), glucose, and xylose (Governo et al., 2004; Rafaïdeen et al., 2019; Moggia et al., 2020; Abdelrahim et al., 2023) on various kinds of electrodes. In addition, glucose belongs to the group of biosourced molecules that can undergo either oxidation or reduction reactions, enabling the production of value-added products at both the anode and cathode of an electrosynthesis cell—a rare occurrence (Pintauro et al., 1984; Möhle et al., 2018; Faverge et al., 2023).

The electro-oxidation of glucose to GA (and also glucaric acid) has been widely studied in strongly alkaline media on mono- or pluri-metallic platinum-group-metal-based catalysts (Pintauro et al., 1984; Largeaud et al., 1995; Beden et al., 1996; Tominaga et al., 2007; Hermans et al., 2011; Mougenot et al., 2011; Yan et al., 2014; Möhle et al., 2018; Rafaïdeen et al., 2019; Moggia et al., 2020; Abdelrahim et al., 2023; Faverge et al., 2023; Neha et al., 2023) and/or transitions metals, such as copper (Moggia et al., 2020) and nickel (Cardoso de Sá et al., 2014; Hwang et al., 2018; Abdelrahim et al., 2023). The former group of catalysts allows oxidation of glucose with relatively good selectivity toward GA at potentials below 1.0 V vs. reversible hydrogen electrode (RHE); however, platinum-group metals are very expensive and/or belong to the list of critical/strategic raw materials (Bobba et al., 2020). The latter group of catalysts allows glucose electro-oxidation at high potentials (exceeding 1.0 V vs. RHE) but often at the expense of the faradaic efficiency and selectivity toward GA or glucaric acid (Sanghez de Luna et al., 2023).

TEMPO or 2,2,6,6-tetramethylpiperidine-1-oxyl may allow some opportunities as an electrochemical catalyst to achieve selective electro-oxidation of glucose to gluconate. Indeed, TEMPO is a stable radical that exhibits useful redox properties and enables high selectivity during the oxidation of primary alcohols to aldehydes or carboxylic acids (Zhao et al., 2005; Omri et al., 2019; Vedovato et al., 2020). It has also been shown to be effective in the oxidation of various polysaccharides, including cellulose, sucrose, and starch (Pierre et al., 2017). TEMPO as a catalyst for selective electro-oxidation of glucose could also promote the use of graphite electrodes while avoiding metal-based electrodes.

Therefore, the present work focuses first on the electro-oxidation of glucose on graphite felt in an alkaline medium (K_2_CO_3_ aqueous electrolyte with pH = 12) in the presence of TEMPO as a homogeneous catalyst. The objective here is to demonstrate the feasibility of a continuous electrochemical reactor that allows selective production of GA at the anode simultaneously with hydrogen production at the cathode. To achieve this objective, further optimizations of the experimental conditions, glucose concentration, electrolyte, TEMPO, and cell voltage are performed using a Taguchi design of experiments (DoE) approach to obtain the highest conversion rate, selectivity toward GA, and faradaic efficiency.

## 2 Experimental

TEMPO (98%), D-glucose (>99%), gluconic acid potassium salt (99%), D-fructose (99%), glucaric acid (99%), potassium carbonate (99%), 4-amino-TEMPO (97%), anhydrous toluene (>99%), thionyl chloride (97%), 4-nitrobenzylamine (97%), triethylamine (99%), dicyclohexylcarbodiimide (DCC; 99%), N,N-dimethylformamide (DMF; 99%), monosodium phosphate (99%), and phosphoric acid (99%) were purchased from Sigma Aldrich. Sulfuric acid (>95–97%) (used for the HPLC mobile phase) was purchased from Merck Millipore, and HPLC-grade water (Milli-Q system, Millipore) was used for the reagents and HPLC mobile phase.

**FIGURE 1:**
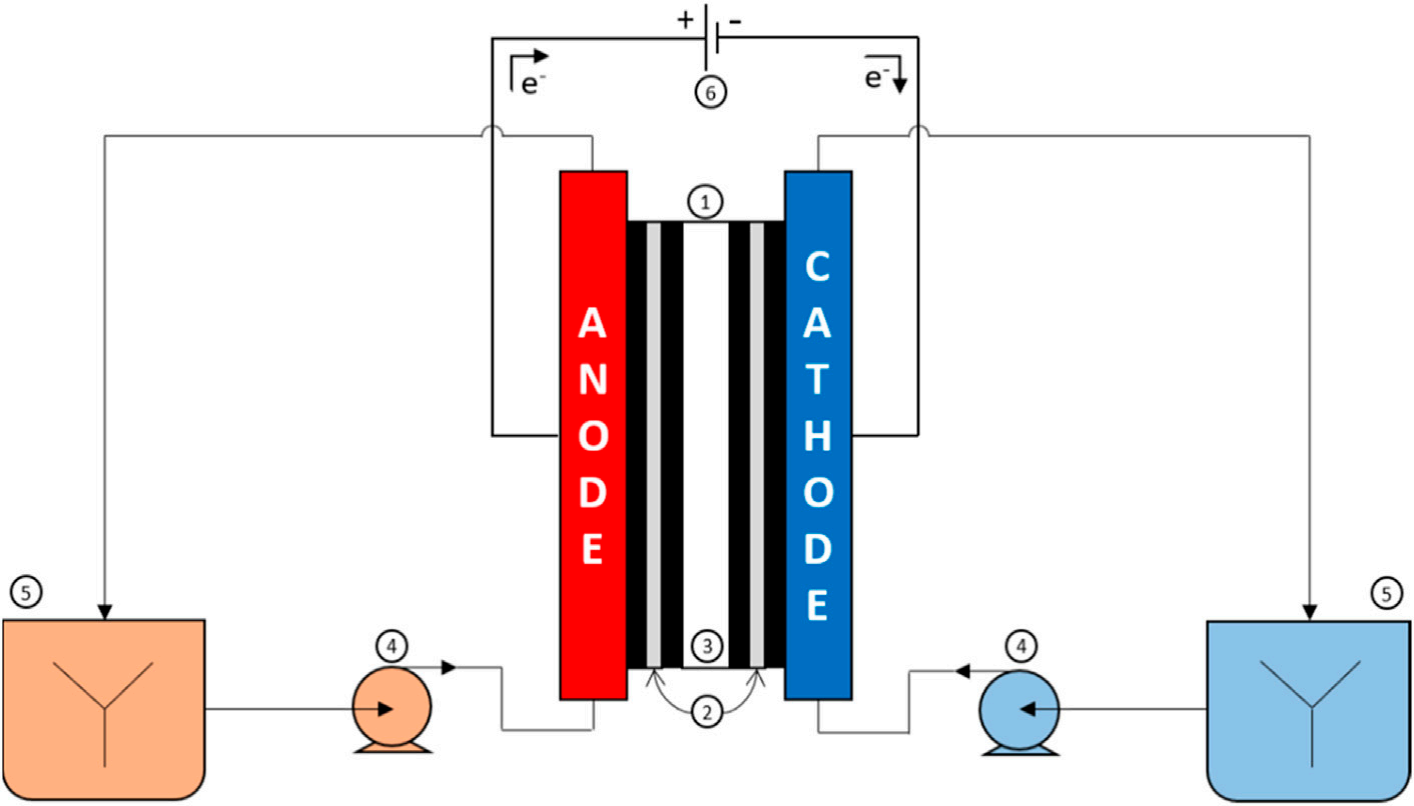
Experimental setup with (1) electrochemical reactor, (2) electrodes, (3) anion-exchange membrane, (4) peristaltic pumps, (5) magnetic stirrers, and (6) potentiostat.

The electrochemical study was performed in an electrochemical reactor made up of a 100 mL cell and a three-electrode setup (an auxiliary electrode, a working electrode and a reference electrode). The electrolyses (Figure 1) were performed using a Micro Flow Cell from ElectroCell (electrode surface area: 10 cm^2^); the anode was made of graphite felt (Goodfellow; thickness: 2 mm, geometric surface area: 44 × 40 mm^2^), the cathode was made of nickel foam (Goodfellow; thickness: 1.6 mm, geometric surface area: 44 × 40 mm^2^), and the reference electrode was Ag/AgCl 3.4 mol L^-1^ KCl inserted in the cathode compartment (1.32 V vs. RHE at pH = 12). The anodic and cathodic compartments were separated by an anion-exchange membrane (AEM) AHA from Eurodia. Constant flow rates were achieved in both compartments using two peristaltic pumps (Masterflex^®^ L/S^®^ Variable-Speed Digital Drive with Remote I/O, 6–600 rpm), and the feeding solutions were non-deaerated and constantly stirred using two magnetic stirrers (Cole-Parmer^®^ SHP-200 Series Undergrad Analog Stirring Hot Plates). The electrolyses were performed at constant anode potentials (chronoamperometries) applied using a Radiometer potentiostat PGP201 through a computer with VoltaMaster 4 software. All potentials are quoted with respect to the RHE.

After electrolysis, the samples were acidified by the addition of 0.1 mL of 0.05 M H_2_SO_4_ such that the pH decreased from 12 to around 1–3 to prevent further degradation of the products under alkaline conditions and to match the pH of the HPLC eluant. The samples for the HPLC analyses were diluted thrice before electrolysis to check the initial concentrations and twice after electrolysis (to achieve uniform product concentrations between 0.1 g L^−1^ and 2 g L^−1^), followed by filtration with a 0.22-µm syringe filter and collection in 2-mL glass screw-top vials.

A Thermo Vanquish HPLC equipped with a Vanquish Split Sampler and an Aminex HPX-87H column (300 × 7.8 mm^2^, 9 µm particle size, 8% cross-linkage, pH: 1–3) was used to analyze the reaction products. Two detectors were used for these analyses, namely, a Vanquish variable-wavelength ultraviolet (UV) detector allowing up to four wavelength acquisitions at the same time (wavelength used: 210 nm) and a refractive index detector (RID; RefractoMax 520). Indeed, glucose cannot be detected with a UV detector. A 10 mmol L^−1^ solution of sulfuric acid was used as a mobile phase under isocratic conditions at a flow rate of 0.6 mL min^−1^. The injection volume was 20 µL, and the column temperature was set to 50°C. The product separation required approximately 20 min, and sample quantification was performed through comparisons with external calibration curves in the range of 0.2–2 g L^−1^ for glucose and fructose and 0.02–2 g L^−1^ for GA and glucaric acid. The linearity of the method was checked using six levels of concentrations each (0.2, 0.5, 0.8, 1.2, 1.5, and 2 g L^−1^ for glucose and fructose; 0.02, 0.1, 0.5, 1, 1.5, and 2 g L^−1^ for GA and glucaric acid). A minimum coefficient of determination (*r* ^2^) of 0.998 was obtained for each calibration curve drawn from both the UV and RI detectors.

Since glucose isomerizes into fructose in an alkaline medium through the Lobry de Bruyn–Van Ekenstein transformation (MacLaurin and Green, 1969; Kokoh et al., 1992) and since this reaction is non-electrochemical in nature, the conversion (Eq. (1)), product yields (Eq. (2)), and product selectivity (Eq. (3)) were calculated as follows by considering only the electrochemical reactions:

$$\text{Reactant conversion }X\;\left(\%\right)=\frac{\text{Amount}\;\text{of}\;\text{glucose}+\text{fructose}\;\text{converted}\;\left(C\;\text{in}\;\text{mole}\right)}{\text{Amount}\;\text{of}\;\text{glucose}+\text{fructose}\;\text{in}\;\text{reactant}\;\left(C\;\text{in}\;\text{mole}\right)}\times 100=\left(1-\frac{C_{\text{glucose}+\text{fructose},t}}{C_{\text{glucose}+\text{fructose},0}}\right)\times 100,$$ (1)

$$\text{Product yield }Y\;\left(\%\right)=\frac{\text{Amount}\;\text{of}\;\text{product}\;\text{formed}\;\left(C\;\text{in}\;\text{mole}\right)}{\text{Amount}\;\text{of}\;\text{glucose}+\text{fructose}\;\text{in}\;\text{reactant}\;\left(C\;\text{in}\;\text{mole}\right)}\times 100=\left(\frac{C_{\text{product},t}}{C_{\text{glucose}+\text{fructose},0}}\right)\times 100,$$ (2)

$$\text{Product selectivity }S\;\left(\%\right)=\frac{\text{Amount}\;\text{of}\;\text{product}\;\left(C\;\text{in}\;\text{mole}\right)}{\text{Amount}\;\text{of}\;\text{glucose}+\text{fructose}\;\text{converted}\;\left(C\;\text{in}\;\text{mole}\right)}\times 100=\left(\frac{C_{\text{product},t}}{C_{\text{glucose}+\text{fructose},0}-C_{\text{glucose}+\text{fructose},t}}\right)\times 100=\frac{Y}{X}.$$ (3)

The faradaic efficiency (FE) is used to determine the amount of electricity utilized for an electrochemical reaction and is calculated as follows:

$$FE=\frac{n_{p}\nu_{e}F}{\nu_{p}Q},\text{with }Q=\int_{0}^{t}\text{Idt},$$ (4)

where $n_{p}$ is the number of moles of the product p obtained when charge *Q* is consumed, $\nu_{e}$ is the number of electrons involved in the electrochemical reaction, $F=96,485\;C\;{\text{mol}}^{-1}$ is the Faraday constant, $\nu_{p}$ is the stoichiometric coefficient of the product obtained, and Q is the charge consumed.

The electrochemical reactions of glucose to gluconate (Eq. (5)) (with $\nu_{e}$ = 2 and $\nu_{p}$ = 1) and glucose to glucarate (Eq. (6)) (with $\nu_{e}$ = 6 and $\nu_{p}$ = 1) are as follows:

$$C_{6}H_{12}O_{6}+{3\text{OH}}^{-}\to C_{6}H_{11}O_{7}^{-}+{2e}^{-}+{2H}_{2}O$$ (5)

$$C_{6}H_{12}O_{6}+{7\text{OH}}^{-}\to C_{6}H_{9}O_{8}^{-}+{6e}^{-}+{5H}_{2}O$$ (6)

## 3 Results and discussion

### 3.1 Preliminary studies

It is well-known that glucose undergoes degradation reactions in non-deaerated alkaline media (Bamford and Collins, 1950). Because the electrolysis measurements were performed over 5 h, the aging of a glucose solution was first studied under conditions close to those used for the electrolysis measurements (graphite felt, 0.028 mol L^-1^ glucose, 0.2 mol L^-1^ K_2_CO_3_, 20%_mol,glucose_ TEMPO, and open-circuit potential). HPLC measurements were performed after 5 h of circulation in the electrolysis cell, and it was found that ca. 2%–3% of glucose was converted to other compounds (formic acid, glycolic acid, etc.) that were qualitatively detectable but not quantifiable.

**FIGURE 2:**
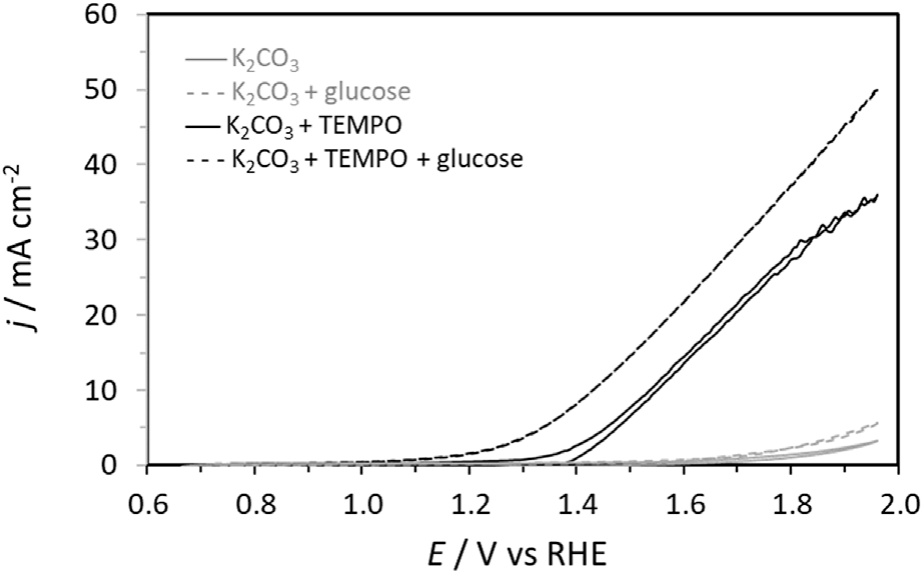
Cyclic voltammetry curves in 0.2 mol L^−1^ K_2_CO_3_ (plain gray line), 0.2 mol L^−1^ K_2_CO_3_ + 0.028 mol L^−1^ glucose (dashed gray line), 0.2 mol L^−1^ K_2_CO_3_ + 0.0056 mol L^−1^ TEMPO (plain black line), and 0.2 mol L^−1^ K_2_CO_3_ + 0.0056 mol L^−1^ TEMPO + 0.028 mol L^−1^ glucose (black dotted line) electrolytes recorded on a graphite felt at 10 mV s^-1^ (*T* = 20°C).

**FIGURE 3:**
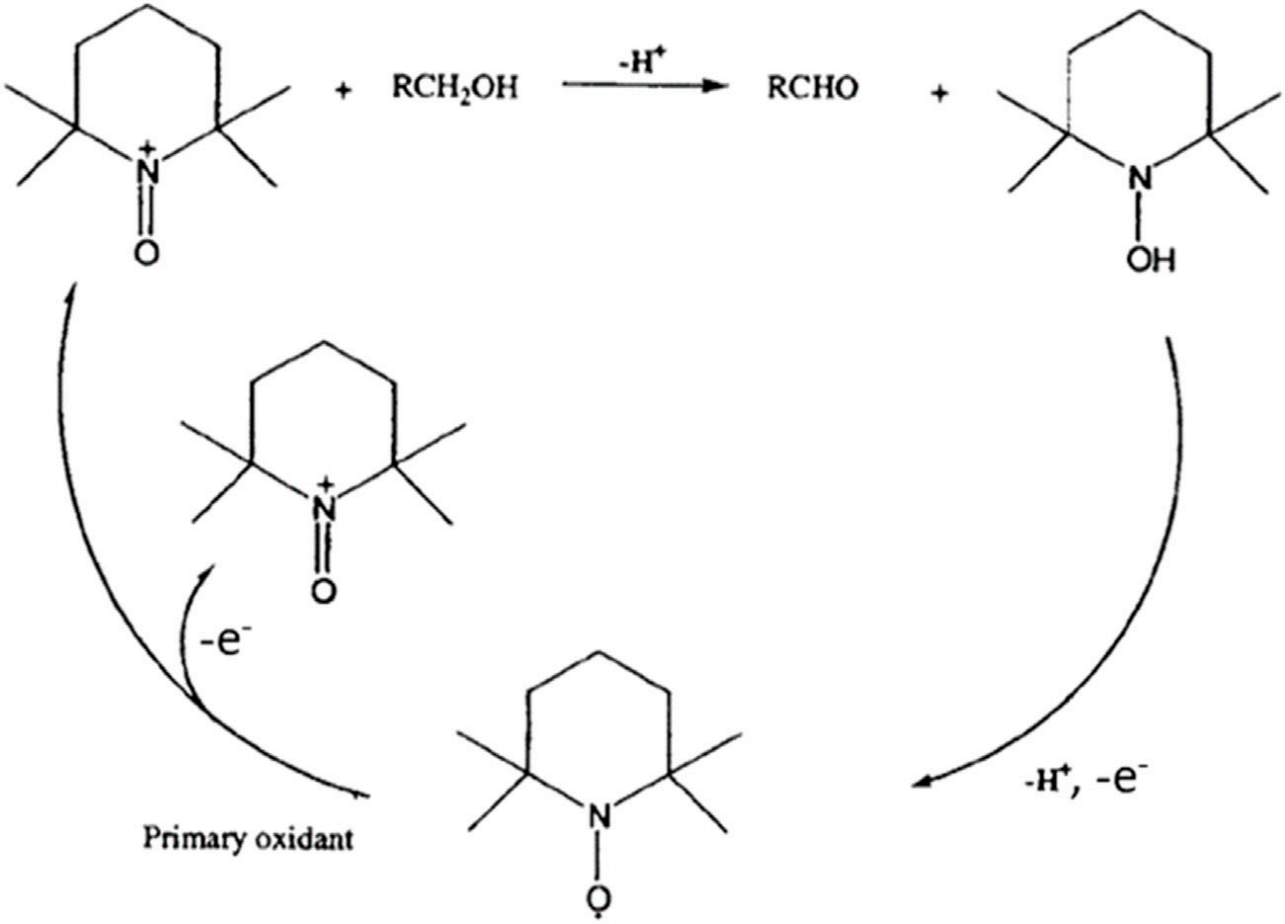
Electrocatalytic cycle of TEMPO oxidation derived from Lindström et al. (2005).

Then, cyclic voltammetry (CV) was performed from 0.66 V to 1.96 V vs. RHE in 0.2 mol L^-1^ K_2_CO_3_ aqueous electrolyte to study the electrochemical activities toward the glucose oxidation reaction (GOR) of graphite felt in the absence and presence of TEMPO (Figure 2). On graphite felt alone, a very low activity was observed for the GOR from ca. 1.60 V vs. RHE as the increase in oxidation current was very small in the presence of glucose compared to those observed in pure electrolytes. The CV curve recorded in the supporting electrolyte in the presence of only TEMPO shows an oxidation wave starting from ca. 1.35 V vs. RHE. From this potential, TEMPO is oxidized to nitrosonium ions; as long as TEMPO exists in this radical form, current can be detected, which reaches a maximum value as the potential increases (explaining the inflexion of the CV) and likely decreases to zero at higher potentials. In the presence of glucose, much higher currents are recorded from ca. 1.20 V vs. RHE, indicating that glucose is catalytically oxidized. These observations translate to the efficiency of oxidation of glucose by TEMPO. At these potentials, TEMPO is oxidized into nitrosonium ions (Figure 3), which can then oxidize a chemical function of a glucose molecule (primary alcohol, secondary alcohol, or anomeric function). Once glucose is oxidized by the nitrosonium ion form of TEMPO, the nitrosonium ions are reduced to a hydroxylamine that can be further re-oxidized on the electrode to the original form of TEMPO to participate in the electrocatalytic cycle. Figure 3 shows an electrochemical mechanism derived from the catalytic cycle proposed by Lindström et al. (2005), with the transfer of two electrons for an oxidation reaction.

Once the activity of TEMPO for the GOR is evidenced, it is necessary to determine the products formed as well as the selectivity and FE of the reaction toward the target product (GA). Here, the determination of the FE is based on the hypotheses that (i) all oxidized TEMPO molecules are consumed to oxidize glucose completely into products and (ii) the contribution of the graphite felt to the glucose electro-oxidation process is negligible. The first hypothesis is supported by the fact that 100% FE toward GA has been achieved under different conditions (Table 3). If a portion of the oxidized TEMPO is used for reactions that do not involve glucose, then 100% FE cannot be achieved. The second hypothesis seems reasonable since no glucose oxidation current is recorded on the carbon felt below 1.6 V vs. RHE (Figure 3).

Chronoamperometry measurements were performed to accumulate the products and determine the product distribution by HPLC. The product distribution can vary depending on the concentrations of glucose, TEMPO, and K_2_CO_3_ as well as the applied anode potential. These parameters were then considered in the study of long-term chronoamperometric measurements. The reference electrolysis experiments were carried out for 5 h in 0.2 mol L^−1^ K_2_CO_3_ electrolyte containing 0.028 mol L^−1^ of glucose and 0.0056 mol L^−1^ of TEMPO (corresponding to a TEMPO/glucose ratio of 20 mol%) with an applied anode potential of 1.300 V vs. RHE and a flowrate of 100 mL min^−1^.

**FIGURE 4:**
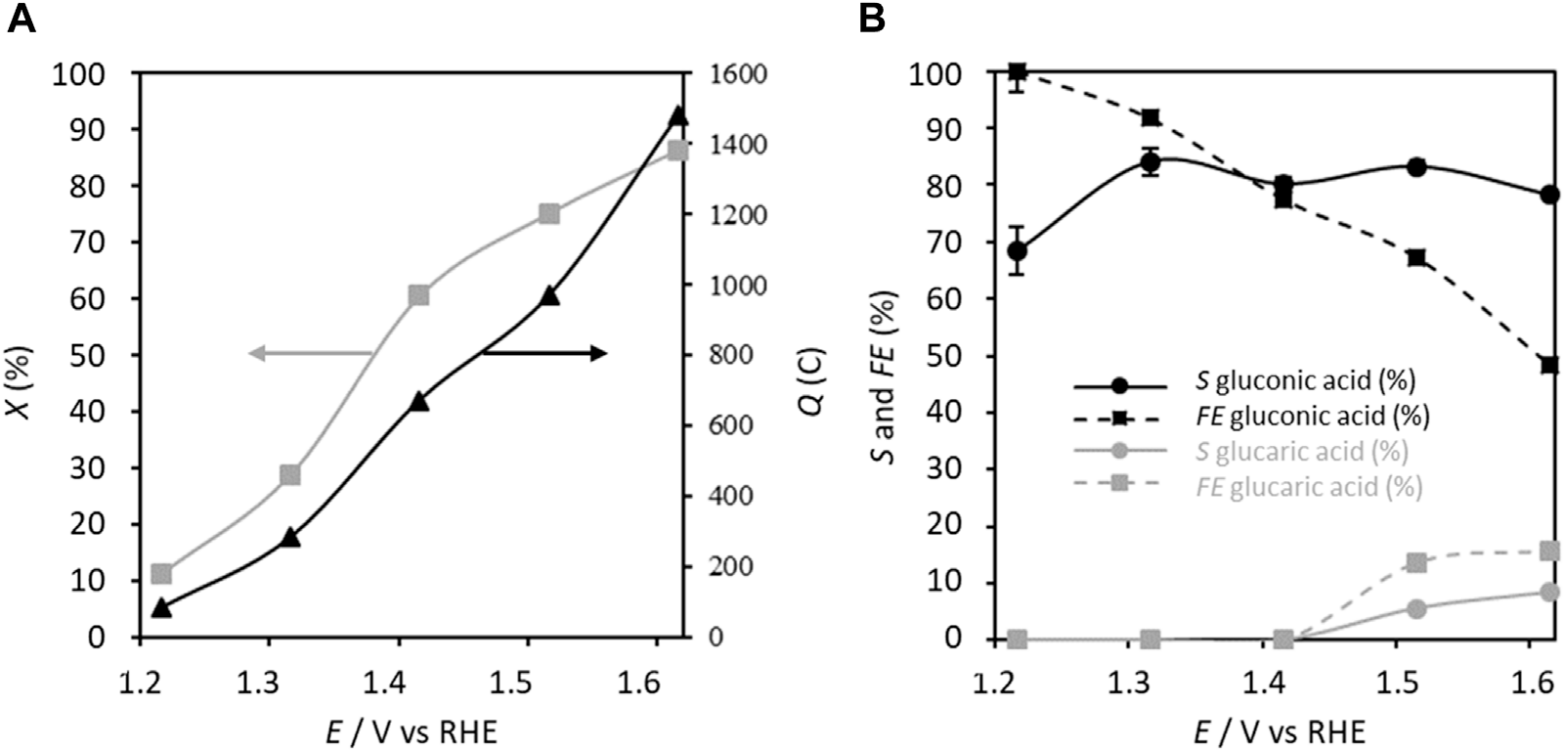
**(A)** Conversion rate *X* and electricity consumed *Q* as well as **(B)** selectivity *S* and faradaic efficiency *FE* in gluconic acid as functions of the applied anode potential; *C*_glucose_ = 0.028 mol L^−1^, *C*_TEMPO_ = 0.0056 mol L^−1^ (20% mol/glucose), *C*_K2CO3_ = 0.2 mol L^−1^, electrolysis time = 5 h, and flow rate = 100 mL min^−1^. The counter electrode is a nickel foam of size 40 × 44 mm^2^ and thickness 1.6 mm (*T* = 20°C).

This configuration of parameters involving an electric charge of 283.2 C resulted in a conversion rate of ca. 28.9%, selectivity toward GA of 84.1%, and FE of 91.8% after 5 h of electrolysis (Figure 4). At this potential, it is unlikely that the oxygen evolution reaction (OER) is significant. Moreover, the CV curve of the carbon felt modified in the presence of only TEMPO in the supporting electrolyte (Figure 3) does not show a significant current density at 1.300 V vs. RHE, and the very small current density observed is likely attributable to the oxidation of TEMPO. Therefore, the FE of 91.8% for GA production may be due to the formation of some byproducts detected by HPLC (formic acid, glycolic acid, and oxalic acid due to alkaline degradation and other products not identified) that are not quantifiable.

Increasing the applied anode potential from 1.32 V to 1.62 V vs. RHE leads to monotonous increases of the electric charge and conversion rate to 1479 C and 86.2%, respectively, at 1.6 V vs. RHE, while the selectivity toward GA decreases slightly to 78.2% and FE drops to 48.4%. Concurrently, for potentials higher than 1.42 V vs. RHE, glucaric acid is detected, reaching a selectivity of 8.4% and FE of 15.6% at 1.62 V vs. RHE. The global FE and selectivity considering both GA and glucaric acid reach 64% and 86.6%, respectively. TEMPO is normally used for its ability to activate primary alcohol oxidation into aldehyde (Lindström et al., 2005). However, under the present experimental conditions, the anomeric function is preferentially oxidized in the presence of TEMPO to form gluconic acid from 1.22 V vs. RHE onwards. The formation of glucaric acid at potentials exceeding 1.42 V vs. RHE proceeds through the oxidation of the C1 alcohol group of GA into a carboxylic acid to form glucaric acid; this is attributable to either a potential effect or catalysis by the nitrosium ion form of TEMPO that is formed more rapidly and at higher amounts at higher potentials. In both cases, it appears that GA becomes a reactive intermediate that can be transformed to glucaric acid. The decrease of the FE for GA production at high anode potentials is due to the formation of not only glucaric acid but also other byproducts detected by HPLC (formic acid and glycolic acid from alkaline degradation and other products not identified) that are not quantifiable (signals below the quantification limit), in addition to the competition with the OER.

**FIGURE 5:**
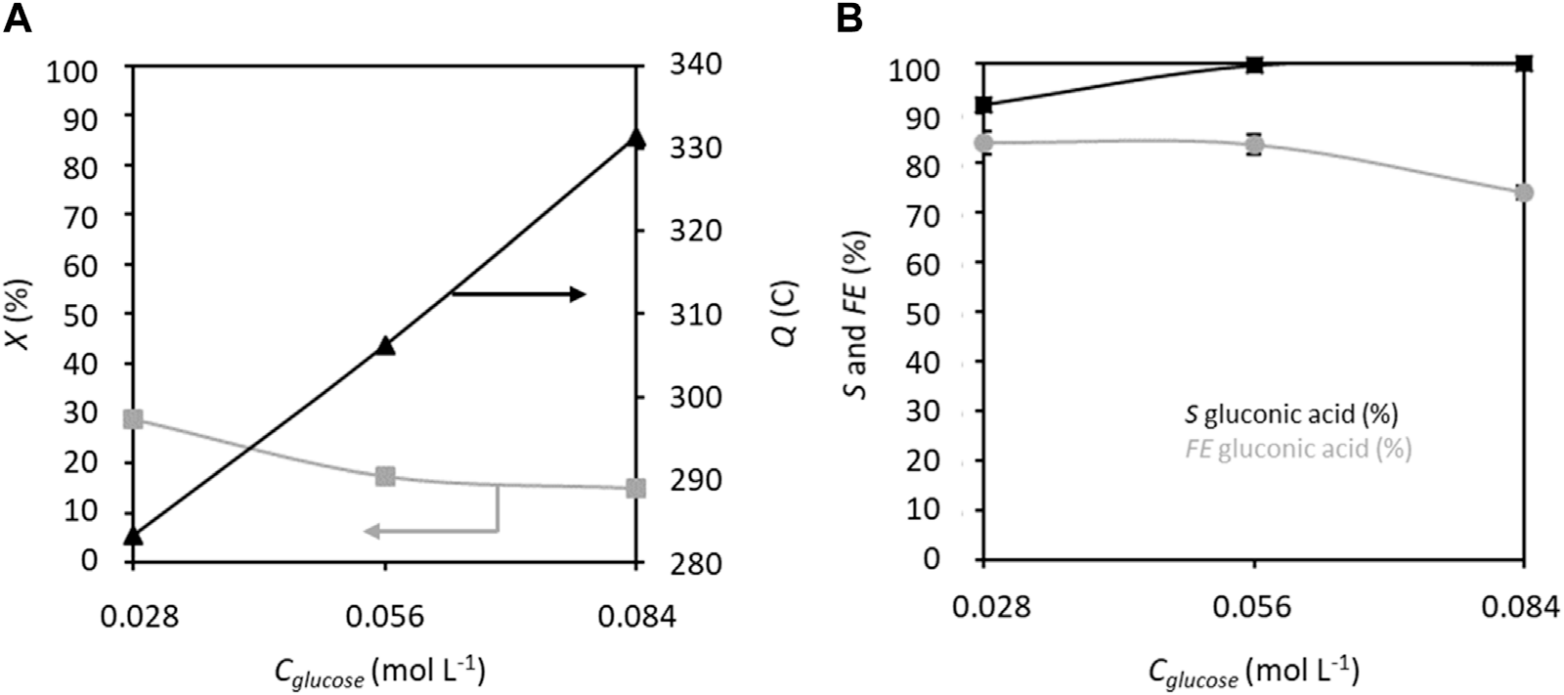
**(A)** Conversion rate *X* and electricity consumed *Q* as well as **(B)** selectivity *S* and faradaic efficiency *FE* in gluconic acid as functions of glucose concentration; *C*_TEMPO_ = 20% mol/glucose, *C*_K2CO3_ = 0.2 mol L^−1^, applied anode potential = 1.32 V vs. RHE, electrolysis time = 5 h, and flow rate = 100 mL min^−1^ (*T* = 20°C). The counter electrode is a nickel foam of size 40 × 44 mm^2^ and thickness 1.6 mm.

The effects of increasing the glucose concentration from 0.028 to 0.084 mol L^−1^ on the electrochemical performance were assessed at 1.32 V vs. RHE (Figure 5). Although the electrical charge increases from 283.2 C to 331.5 C with this increase in glucose concentration, the conversion rate decreases from 29% to 15%. The increase in electrical charge is only 17% for a 300% increase in glucose concentration, which explains the decrease of the ratio *C*_gluconic,t_/*C*_glucose+fructose,0_ (conversion rate) with increasing concentration. Several explanations can be proposed for this observation, such as saturation of the graphite felt surface by glucose/fructose that limits the access of TEMPO, presence of a limited number of active surface sites for reaction, or poisoning of the surface. The selectivity decreases slightly for glucose concentrations between 0.028 and 0.056 mol L^−1^ (*S* around 84%) and more drastically for the highest concentration (*S* = 74%). However, the FE improves with increase in glucose concentration, starting at 91.8% for 0.056 mol L^−1^ glucose and reaching 100% for 0.084 mol L^−1^ glucose. This means that 100% of the electricity is consumed toward oxidizing glucose into GA at high glucose concentrations. The discrepancy between the decreasing selectivity and increasing FE with increasing glucose concentration can be attributed to the products formed by non-electrochemical degradation reactions (ca. 2%–3% for 0.028 mol L^−1^ glucose in 0.2 mol L^−1^ K_2_CO_3_ and 20% mol TEMPO) and unidentifiable side products, which may decrease the selectivity without altering the FE. It has indeed been shown in a previous work that higher glucose concentrations exhibit higher degradation kinetics in alkaline media (Rafaïdeen et al., 2020).

**FIGURE 6:**
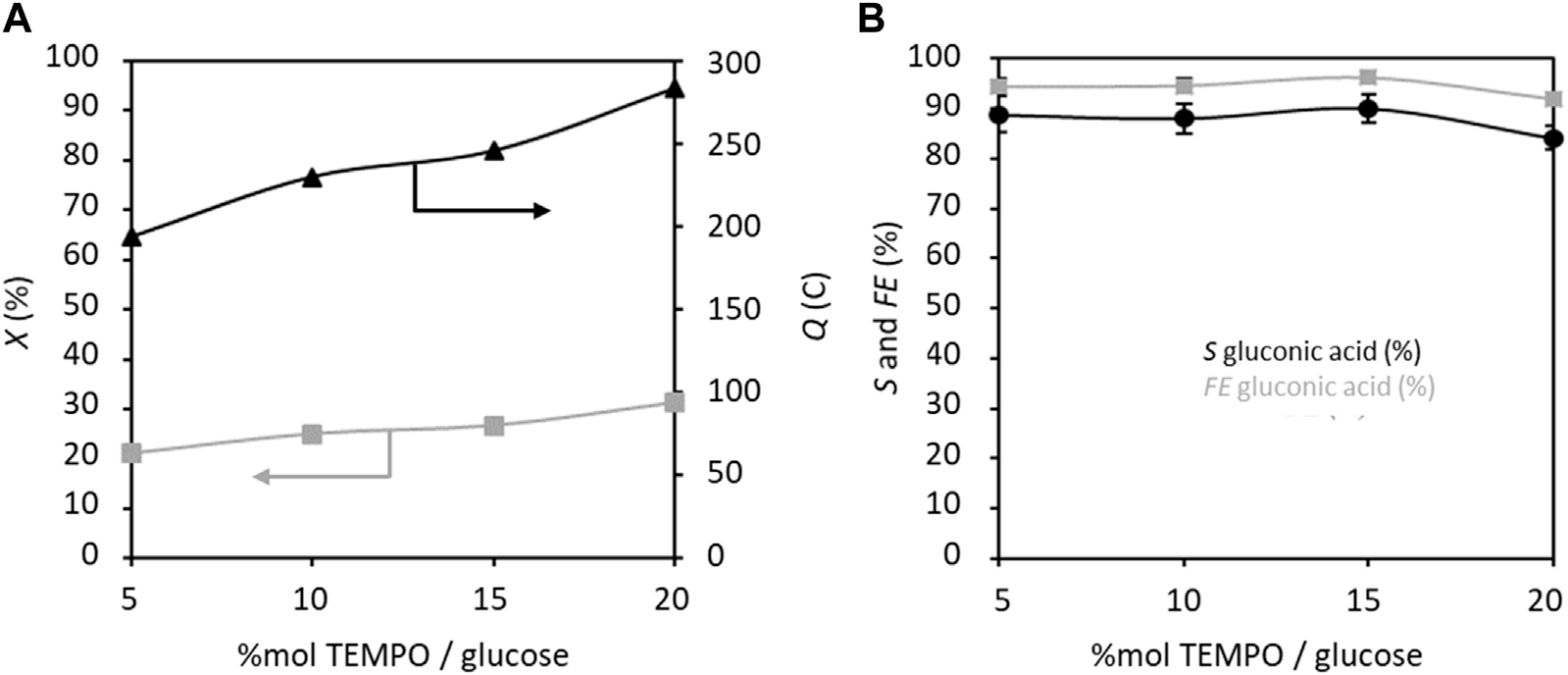
**(A)** Conversion rate *X* and electricity consumed *Q*, and **(B)** selectivity *S* and faradaic efficiency *FE* in gluconic acid as functions of TEMPO concentration (% mol/glucose); *C*_glucose_ = 0.028 mol L^−1^, C_K2CO3_ = 0.2 mol L^−1^, applied anode potential = 1.32 V vs. RHE, electrolysis time = 5 h, and flow rate = 100 mL min^−1^ (*T* = 20°C). The counter electrode is a nickel foam of size 40 × 44 mm^2^ and thickness 1.6 mm.

Next, the effects of varying the proportion of TEMPO from 5%_mol glucose_ to 20%_mol glucose_ on the GOR were studied (Figure 6). Here, both electrical charge and conversion rate follow the same trend and increase with the proportion of TEMPO. The selectivity and FE also remain almost stable over the concentration range of TEMPO and seem to decrease slightly at higher concentrations. It is worth noting that a large amount of TEMPO is not required for achieving high selectivity and FE (88% and 94.4%, respectively, for 5% TEMPO). Again, an FE value below 100% indicates that other coproducts are electroformed that are detectable by HPLC but not identifiable, in addition to formation of non-electrochemical degradation products, both with very low selectivity values.

**FIGURE 7:**
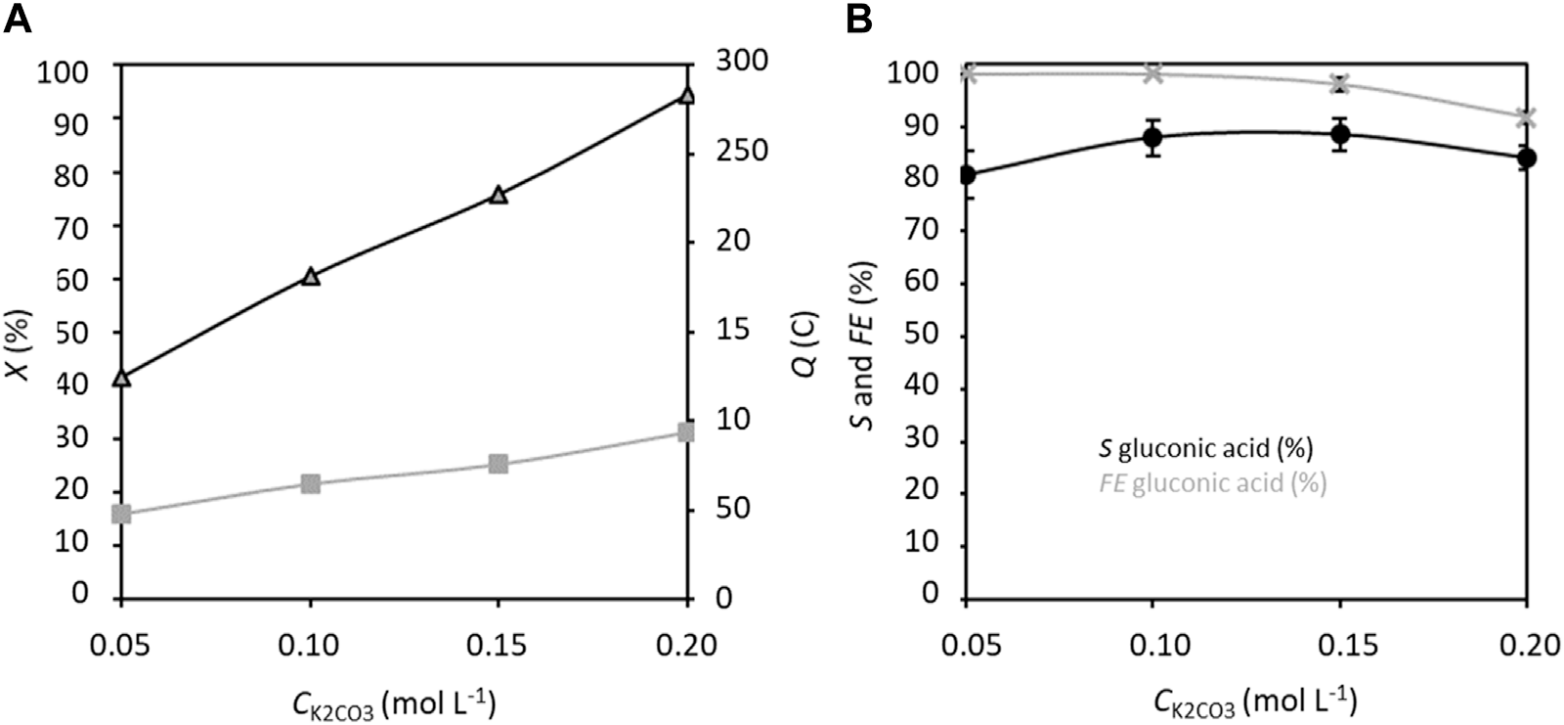
**(A)** Conversion rate *X* and electricity consumed *Q*, and **(B)** selectivity *S* and faradaic efficiency *FE* in gluconic acid as functions of *C*_K2CO3_; *C*_Glucose_ = 0.028 mol L^−1^, *C*_TEMPO_ = 0.0056 mol L^−1^ (20% mol/glucose), applied anode potential = 1.32 V vs. RHE, electrolysis time = 5 h, and flow rate = 100 mL min^−1^ (*T* = 20°C). The counter electrode is a nickel foam of size 40 × 44 mm^2^ and thickness 1.6 mm.

Lastly, the effects of K_2_CO_3_ concentration variation in the range of 0.05–0.20 mol L^−1^ (Figure 7) were studied. The conversion rate increases obviously with K_2_CO_3_ concentration due to the increasing conductivity. The selectivity increases for K_2_CO_3_ concentrations from 0.05 to 0.10 mol L^−1^, before stabilizing at ca. 90% between 0.10 and 0.15 mol L^−1^ K_2_CO_3_, with a faradaic efficiency very close to 100%. The competition between the electroconversion of glucose to GA and chemical degradation of glucose in an alkaline medium appears to limit the selectivity to a maximum of 90% after 5 h of measurements. The optimal concentration of K_2_CO_3_ thus seems to be between 0.10 and 0.15 mol L^−1^.

**FIGURE 8:**
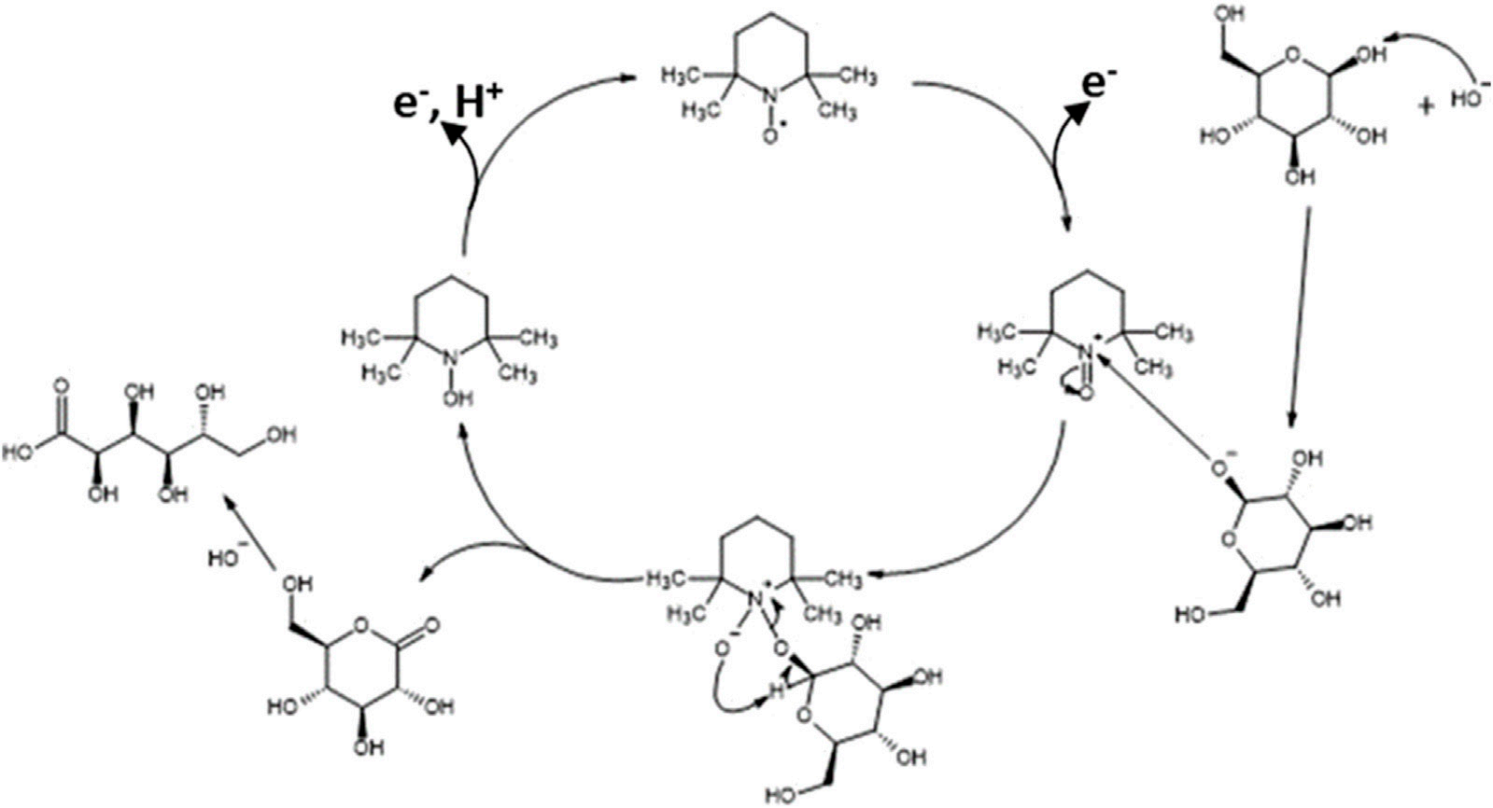
Mechanism of electrocatalytic oxidation of glucose by TEMPO.

In all these experiments, the main reaction product by far is GA, which demonstrates that TEMPO is active for the electrocatalytic oxidation of the anomeric function of glucose. Because the cyclic form of glucose is predominant in the aqueous solution and because it is known that the OH group of the anomeric function is highly reactive to oxidation, the mechanism in Figure 8 is proposed for the oxidation of glucose to GA, which is derived from the mechanism proposed for alcohol oxidation by TEMPO (Rafiee et al., 2015). In this mechanism, the TEMPO molecule is oxidized by transferring two electrons toward the carbon felt electrode; the oxidized form of TEMPO then catalyzes the oxidation reaction of the anomeric function.

### 3.2 Optimization of experimental conditions via DoE

A preliminary study of glucose electro-oxidation on graphite felt in the presence of TEMPO showed that the concentrations of the electrolyte, glucose, and TEMPO as well as the anode potential are important parameters influencing the conversion rate, selectivity, and FE of the reaction. To optimize the experimental conditions and determine the best configurations of these parameters that can achieve the highest conversion rate, selectivity, and FE for glucose electro-oxidation into GA, a DoE approach was used based on the L_9_ (3^4^) orthogonal array of the Taguchi table (Table 1). Taguchi’s method is a powerful tool for the design of a high-quality system that provides a simple, an efficient, and a systemic approach to optimization, quality, and cost (Ross, 1988) while diminishing the number of experiments. Here, the study of four parameters with three levels each necessitates 3^4^ experiments (81 experiments) in the full-factorial DoE. This number can be decreased to nine using the L_9_ (3^4^) orthogonal array of the Taguchi table if the interactions between the parameters are neglected.

**TABLE 1:** L_9_ orthogonal array of the Taguchi method.

Experiment	Parameters and levels			
	A	B	C	D
1	1	1	1	1
2	1	2	2	2
3	1	3	3	3
4	2	1	2	3
5	2	2	3	1
6	2	3	1	2
7	3	1	3	2
8	3	2	1	3
9	3	3	2	1

A: glucose concentration, B: K_2_CO_3_ concentration, C: TEMPO concentration, and D: applied anode potential; the levels 1, 2, and 3 correspond to the low, medium, and high values of the parameters, respectively (see Table 2).

Each of the nine experiments was performed for the four parameters set to the levels given in Table 1 (L_9_ (3^4^) orthogonal array of Taguchi), and their corresponding values are noted in Table 2. Table 3 gives the matrix of experiments as well as the responses (conversion rate, FE, and selectivity) obtained for each experiment with the parameters set to appropriate values.

**TABLE 2:** Values of each of the parameters for all levels.

	Level 1	Level 2	Level 3
A: [Glucose] (mol L^-1^)	0.028	0.056	0.084
B: [K_2_CO_3_] (mol L^-1^)	0.05	0.1	0.2
C: [TEMPO] (%_mol glucose_)	5	10	15
D: E (V vs. RHE)	1.32	1.42	1.52

**TABLE 3:** Reactant conversion rate *X*, chemical selectivity *S*, and faradaic efficiency *FE* calculated from the nine experiments of the Taguchi L_9_ method.

Experiment	*C*_glucose_ mol L^−1^	*C*_K2CO3_ mol L^−1^	% TEMPO	*E* V vs. RHE	*X* (%)	*FE* (%)	*S* (%)
1	0.028	0.05	5	1.32	11.6	100	76.7
2	0.028	0.1	10	1.42	31.1	98.3	87.8
3	0.028	0.2	15	1.52	78.9	71.0	83.8
4	0.056	0.05	10	1.52	24.8	100	87.6
5	0.056	0.1	15	1.32	16.6	100	74.9
6	0.056	0.2	5	1.42	33.7	92.0	85.5
7	0.084	0.05	15	1.42	14.9	100	73.5
8	0.084	0.1	5	1.52	27.6	100	80.1
9	0.084	0.2	10	1.32	18.1	100	73.3
Average					28.6	95.7	80.5

The results in Table 3 make it possible to theoretically calculate the response Y of the system for any configuration of factors from the effects E_Ai_ of the parameters using the following equations:

$$Y=M+E_{C_{\text{glucose}}}+E_{C_{K2\text{CO}3}}+E_{\%\text{TEMPO}}+E_{E},$$ (7)

$$\text{With},{\;E}_{A_{i}}=\frac{\sum R_{A_{i}}}{3}-\bar{R},$$ (8)

where M is the general average of the responses, E_Ai_ is the effect of parameter A at level i, R_Ai_ is the measured response for parameter A at level i, and $\bar{R}$ is the average value of the responses.

**FIGURE 9:**
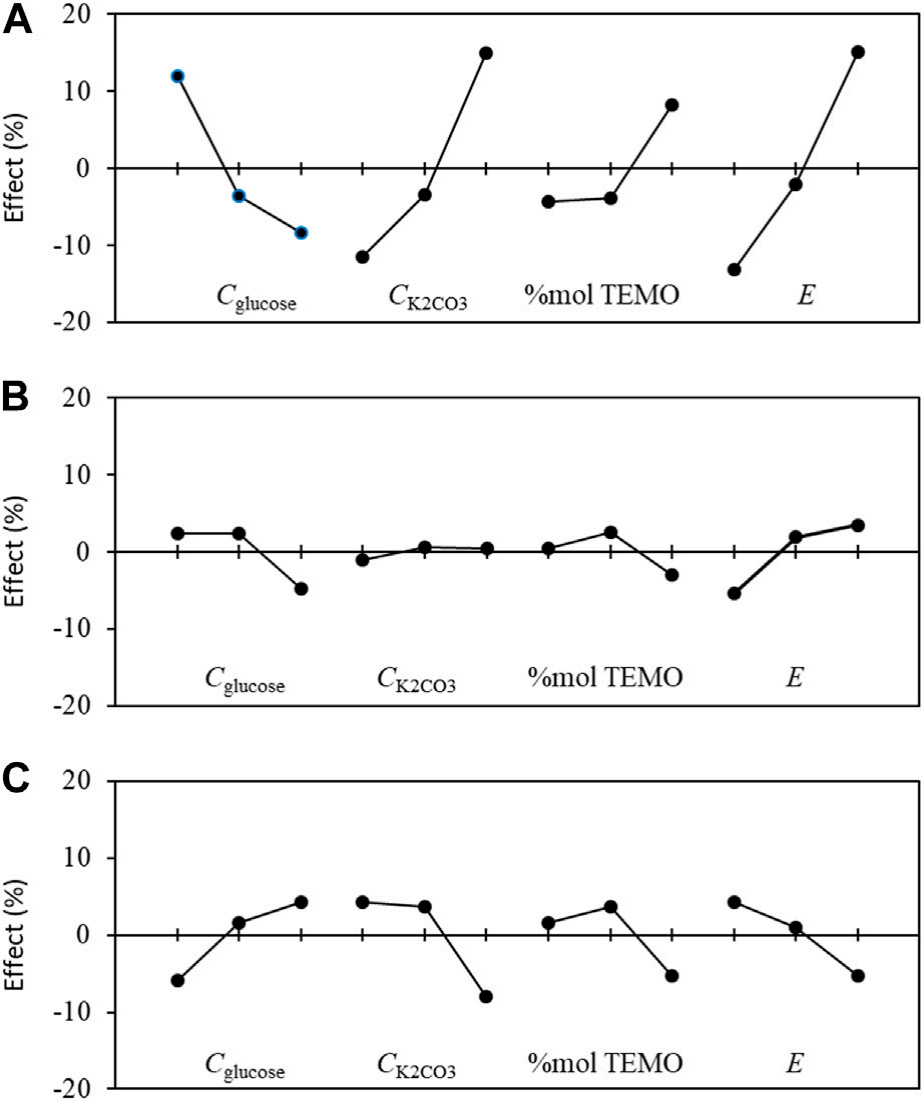
Graphs of the mean effects of the experimental parameters, as determined from Eq. (5) on the **(A)** conversion rate *X*, **(B)** selectivity *S*, and **(C)** faradaic efficiency *FE* toward gluconic acid.

The effect graphs in Figure 9 show the influences of the levels of each parameter on different responses, i.e., conversion rate *X*, chemical selectivity S, and faradaic efficiency FE%. From these graphs, it is possible to optimize the operating conditions to obtain the best reactant conversion rate, chemical selectivity, and FE.

Considering the optimization of the conversion rate *X* (Figure 9A) first, the best parameter configurations are *C*_glucose_ at level 1 (0.028 mol L^−1^), C_K2CO3_ at level 3 (0.2 mol L^−1^), %_mol TEMPO_ at level 3 (15%_mol/glucose_, 0.0042 mol L^−1^), and *E* at level 3 (1.52 V vs. RHE). This experiment is within the DoE and leads to values (both experimental and theoretical) of 78.9% for the conversion rate *X*, 81.6% for the selectivity *S*, and 76.9% for the faradaic efficiency *FE*. Now, considering the optimization of selectivity *S* (Figure 9B), the best parameter configurations are *C*_glucose_ at level 1 (0.028 mol L^−1^), *C*_K2CO3_ at level 2 (0.1 mol L^−1^), %_mol TEMPO_ at level 2 (10%_mol/glucose_, 0.0028 mol L^−1^), and *E* at level 3 (1.52 V vs. RHE); this experiment is out of the DoE and can therefore serve as a confirmation to verify the accuracy of the DoE. Accordingly, the experimental values were *X*_exp_ = 36.4, *S*_exp_ = 87.8%, and *FE*_exp_ = 70.1%, whereas the theoretical values were *X*_theo_ = 48.3%, *S*_theo_ = 89.4%, and *FE*_theo_ = 91.9%. Lastly, for optimization of the faradaic efficiency *FE* (Figure 9C), the best parameter configurations are *C*_Glucose_ at level 3 (0.084 mol L^−1^), *C*_K2CO3_ at level 1 (0.05 mol L^−1^), %_mol TEMPO_ at level 2 (10%_mol/glucose_, 0.0084 mol L^−1^), and *E* at level 1 (1.32 V vs. RHE). This experiment is also out of the DoE. Using these experimental conditions, *X*_exp_ = 8.3%, *S*_exp_ = 78.5%, and *FE*_exp_ = 100%, whereas the corresponding theoretical values are *X*_theo_ = −8.4%, *S*_theo_ = 71.7%, and *FE*_theo_ = 112.3%.

For the three responses (conversion rate, selectivity, and FE), the theoretical and experimental values obtained in the confirmation experiments are different, which indicates interactions between the parameters. However, the variations of the theoretical values have the same trends as those of the experimental values. Moreover, the differences are lower for selectivity than for the other responses. However, adding one or two independent interactions in the DoE will necessitate performing at least 27 or all 81 experiments (corresponding to the full-factorial DoE), respectively, to respect both the degree of liberty and orthogonality criteria (Ross, 2017). Since the theoretical and experimental values of each of the responses follow similar trends, the DoE allows the following track. The optimization of the *FE* is at the expense of the conversion rate and selectivity. Therefore, the experiment within the DoE with *C*_glucose_ = 0.028 mol L^−1^, *C*_K2CO3_ = 0.2 mol L^−1^, %mol TEMPO = 15%_mol/glucose_ (0.0042 mol L^−1^), and *E* = 1.52 V vs. RHE that results in *X*_exp_ = 78.9%, *S*_exp_ = 81.6%, and *FE*_exp_ = 76.9% seems to be the best compromise in terms of optimization of all responses. However, the user may choose a different set of parameter configurations according to the response that they consider the most important.

## 4 Conclusion

TEMPO is generally used as the catalyst for selective oxidation of primary alcohols toward aldehydes. Herein, TEMPO is successfully uses as the homogeneous catalyst for selective electro-oxidation of glucose. The effects of different experimental parameters (glucose and K_2_CO_3_ concentrations, molar ratio of TEMPO with respect to glucose, and applied anode potential) on the glucose conversion rate, selectivity, and faradaic efficiency toward GA were studied. Optimizations of the experimental conditions were also performed using a DoE approach derived from the L_9_ (3^4^) Taguchi array to achieve high selectivity of 88% toward GA with a faradaic efficiency of 70% for a conversion rate of 36.4% (*C*_glucose_ = 0.028 mol L^−1^, *C*_K2CO3_ = 0.1 mol L^−1^, TEMPO = 10%_mol/glucose_ = 0.0028 mol L^−1^, and *E* = 1.52 V vs. RHE). The glucose conversion rate can be further increased to 79%, with the faradaic efficiency and selectivity remaining at approximately 77% and 82%, respectively (*C*_glucose_ = 0.028 mol L^−1^, *C*_K2CO3_ = 0.2 mol L^−1^, TEMPO = 15%_mol/glucose_ = 0.0042 mol L^−1^, and *E* = 1.52 V vs. RHE). About 10% glucaric acid conversion can also be obtained when the applied anode potential is above 1.52 V vs. RHE.

## Data Availability

The raw data supporting the conclusions of this article will be made available by the authors without undue reservation.
